# Perceived cognitive deficits are associated with diabetes self-management in a multiethnic sample

**DOI:** 10.1186/s40200-017-0289-3

**Published:** 2017-02-15

**Authors:** Heather Cuevas, Alexa Stuifbergen

**Affiliations:** The University of Texas, Austin School of Nursing, 1710 Red River, Austin, TX 78701 USA

**Keywords:** Type 2 diabetes, Cognitive function, Self-management, Executive function, Quality of life

## Abstract

**Background:**

People with diabetes have almost twice the risk of developing cognitive impairment or dementia as do those without diabetes, and about half of older adults with diabetes will become functionally disabled or cognitively impaired. But diabetes requires complex self-management: patients must learn about the implications of their disease; manage their diets, physical activity, and medication; and monitor their blood glucose. Difficulties with cognition can hinder these activities.

**Methods:**

The purpose of this study was to explore perceptions of cognitive ability in a multiethnic sample of persons with type 2 diabetes (T2DM). One hundred twenty participants completed surveys assessing perceived memory, executive function, diabetes self-management, and quality of life. Scores on the surveys were examined along with hemoglobin A1C levels and demographics.

**Results:**

Scores for executive function were positively associated with self-reports of dietary adherence and blood glucose monitoring. Perceived memory ability was a significant predictor of quality of life, and executive function was a significant predictor of A1C.

**Conclusions:**

Patients’ perceptions of their cognitive difficulties may assist health care providers in detection of patients’ deficiencies in performing diabetes self-management tasks. The relationships between cognitive difficulties and self-management found in this descriptive study suggest that research on the processes leading to cognitive changes in T2DM is needed, as are studies on how those processes affect diabetes self-management.

## Background

Diabetes is a known risk factor for cognitive impairment, owing to mechanisms such as chronic hyperglycemia or hypoglycemia, insulin resistance or hyperinsulinemia, oxidative stress, and build-up of beta-amyloid protein in the brain [1]. People with type 2 diabetes (T2DM) have cognitive impairment at rates that are twice as high as those for people without T2DM and having diabetes in midlife is associated with an almost 20% greater decline in cognitive function over 20 years then for those without T2DM [2]. In addition, some studies have found changes in brain structure and brain activation patterns with obesity, insulin resistance, and/or metabolic syndrome before development of overt T2DM [2]. It is often assumed that informing a patient about diabetes self-management skills, including diet, self-monitoring of blood glucose, and medication management, will positively influence glycemic control. Yet it is possible that when patients feel they are experiencing cognitive problems it will affect all these tasks, and little has been done to examine the combined effects of diabetes and cognitive impairment on diabetes self-management—especially in minority groups with a higher prevalence of T2DM and a higher disease burden [3, 4]. Recent examinations of cognitive function and diabetes self-management have been done with small, mostly non-Hispanic white samples, demonstrating a gap in our understanding of diabetes and cognitive function and how diabetes is associated with impairment in specific cognitive domains such as memory and executive function in minority groups [5, 6]. The purpose of this study is to examine the relationships between perceived cognitive function, diabetes self-management, quality of life, and glycemic control in a multiethnic sample.

## Methods

### Design, study population, and data collection

This was a cross-sectional, descriptive correlational study. Over a 5-month period, a convenience sample of participants from 21 to 70 years old with T2DM was recruited from an endocrinology clinic in close proximity to the authors’ university. Recruiting efforts included the placement of flyers in the clinic’s exam rooms and research office as well as face-to-face contact by the PI with potential participants. Potential participants who expressed interest in the study were screened for eligibility by the PI and a member of the clinic’s research staff by asking potential participants questions based on the inclusion/exclusion criteria. Inclusion criteria were as follows: ages from 21 to 70; living with diabetes for at least 1 year; ability to read, speak, and understand English. Because survey responses from participants with dementia can be unreliable, those previously diagnosed with dementia were excluded as well. Patients with hypertension and hyperlipidemia were included. Those with type 1 diabetes or who did not know what type they had were also excluded.

One hundred and eighty-five potential participants were screened. After the inclusion/exclusion criteria were applied, 135 patients remained. These participants received paper copies of the study’s survey instruments as well as an addressed/stamped envelope to return the surveys to the primary investigator. They were advised that return of their questionnaires to the primary investigator signified informed consent. Of them, 122 returned surveys. Two patients who returned the survey were excluded because they indicated that they had been diagnosed with cognitive impairment (Alzheimer’s disease and impairment after a stroke) when asked to list other co-morbidities; the final sample included 120 participating patients.

### Measures

The following survey instruments were used.

#### Perceived memory

The 57-item Multifactorial Memory Questionnaire (MMQ) assesses three dimensions of memory self-appraisal: contentment (feelings about one’s memory), ability (subjective impressions of one’s memory capability), and strategy (reported frequency of use of various memory aids) [7]. The 18 items for contentment are rated on a 5-point scale (0 = strongly agree, 1 = agree, 2 = undecided, 3 = disagree, 4 = strongly disagree), with higher scores suggesting higher contentment about memory ability. Scores range from 0 to 72, with Cronbach’s alpha = 0.95 [7]. For ability, 20 items ask participants to indicate how often specific memory failures have occurred in the past 2 weeks on a 5-point scale (0 = all the time, 1 = often, 2 = sometimes, 3 = rarely, 4 = never). Higher scores reflect better subjective memory ability, with a score range of 0–80 and a Cronbach’s alpha of 0.93. For strategy, 19 items ask participants to rate how often they use certain memory strategies (0 = never, 1 = rarely, 2 = sometimes, 3 = often, 4 = all the time). Scores range from 0 to 76, with alpha = 0.83 [7].

#### Perceived executive function

The Barkley Deficits in Executive Function Scale–Short Form (BDEFS-SF) is used to evaluate executive function in adults ages 18–81 [8]. The instrument shows the types and extent of perceived executive function deficits in daily activities over an extended period of time [8]. Items on the self-report version assess how often participants have engaged in behaviors that represent specific types of executive functioning over the past 6 months: time management, self-organization/problem-solving, self-restraint, self-motivation, and self-regulation of emotion. Items are measured on a 4-point Likert scale ranging from 1 = never or rarely to 4 = very often. The short form contains 20 items and requires 4–5 min to complete. Scores from each subscale are tallied and added to yield a total score. Percentile ranks are computed from the raw scores and compared with norms determined by age. Higher percentile rankings within a subscale represent greater executive functioning deficits in that particular area. Higher percentile scores from the total raw score represent general executive functioning deficits. Internal consistency for the short-form scale is high, with Cronbach’s alpha = 0.92. Pearson’s *r* correlations across subscales have been found to range from 0.55 to 0.80; test-retest reliability, from 0.62 to 0.80 in prior studies [8].

#### Diabetes self-management adherence

The Summary of Diabetes Self Care Activities (SDSCA) measures five self-reported activities in diabetes self-management: self-monitoring of blood glucose, diet, checking one’s feet, exercise, and smoking habits [9]. Participants answer 25 questions about the number of days in the last week on which they have performed specific aspects of diabetes self-management. For example, a participant has the choice to select a number, 0 through 7, in answer to the question, “How many of the last seven days have you checked your blood sugar?” Scores are calculated for each of the five regimen areas; higher scores indicate greater participation in self-management behaviors. Inter-item correlations have been found to range from *r* = 0.20 to *r* = 0.76 for the SDSCA subscales; 4-month test-retest reliability ranges from *r* = -0.05 to 0.78 in prior studies [9].

#### Quality of life

The RAND 36-item Short Form Health Survey (RAND SF-36) was developed as part of a multi-year, multi-site study designed to explain variations in patient outcomes. It measures eight health dimensions commonly included in widely used health surveys: role limitations due to physical problems, social functioning, physical functioning, bodily pain, general mental health, role limitations due to emotional problems, vitality, and general health perceptions. It also includes a single item that indicates perceived change in health [10]. Total scores range from 0 (poorest health) to 100 (best health). The survey’s scales have been shown to have adequate internal consistency, with alphas ranging from 0.72 to 0.94 [11]. Test-retest values ranged from 0.60 to 0.90, with the exception of bodily pain at 0.43. Factor analysis resulted in two higher order factors representing the physical and mental health constructs of the instrument. The subscale of “general health” was used to indicate quality of life for this project.

#### Demographics

Participants also completed a short demographic form that included questions regarding age, ethnicity, A1C level, educational level, medications, and co-morbidities.

### Data analysis

Data were analyzed using the Statistical Package for Social Sciences, Version 22.0 [12]. Descriptive variables were reported (numbers, percentages, means with standard deviations) for all variables. Pearson’s bivariate correlation analysis was used to assess correlations between demographic variables and the survey scores (MMQ, BDEFS-SF, etc.) and between the survey scores themselves. The significance level was set at 0.05. Separate multiple linear regressions were conducted to predict the impact of perceived cognitive function on quality of life and A1C.

## Results

### Demographic characteristics

Table 1 presents the characteristics of the total sample (*n* = 120). Half of the participants were from minority populations. Participants’ mean reported A1C was 8.6% (SD = 1.5). The number of co-morbidities ranged from 0 to 4; 75% had hyperlipidemia; 62.2%, hypertension; 15.6%, hypothyroidism. There were no significant differences in number of co-morbidities between age groups. Fifty-one percent were on oral glucose-lowering medications, 31% on insulin, and 17.5% on a combination of oral medications and insulin. Only 4.2% of the participants were not on any medications, and 1.6% noted the type of medication as “other” and wrote it in as an injectable non-insulin such as liraglutide. Results from the SDSCA show that adherence to diet and exercise was low, with participants averaging only 3 days a week of adherence to a healthy diet and less than 2 days a week of at least 30 min of exercise.

**Table 1:** Participants’ characteristics

	Total sample (*n* = 120)	Non-Hispanic White (*n* = 54)	Hispanic (*n* = 44)	African American (*n* = 21)	Asian (*n* = 1)
Age (years)	53.7 (3.5)	53.7 (14.0)	53.5 (12.7)	52.1 (13.8)	33.0
Gender (% female)	49.2 (*n* = 59)	40.7	50.0	76.2	100
Education %(n)					
High School	15 (18)	14.8 (8)	15.9 (7)	9.5 (2)	100 (1)
Some College	15 (18)	14.8 (8)	18.2 (8)	9.5 (2)	
Associates	16.7 (20)	14.8 (8)	18.2 (8)	19.0 (4)	
College Graduate	36.8 (44)	35.2 (19)	34.1 (15)	47.6 (10)	
Master’s Degree	8 (10)	9.3 (5)	6.8 (3)	9.5 (2)	
Doctoral Degree	8 (10)	11.1 (6)	6.8 (3)	4.8 (1)	
Diabetes duration (years)	6.7 (5.1)	6.4 (4.6)	7.0 (5.2)	7.4 (6.0)	1.0
A1C	8.6 (1.5)	8.6 (1.5)	8.8 (1.6)	8.1 (1.3)	6.0
Number of co-morbidities	1.3 (1.03) range = 0 to 4	1.3 (0.99) range = 1 to 3	1.4 (1.06) range = 1 to 4	1.2 (1.0) range = 1 to 3	0
Days per week of diet adherence	3.28 (1.29)	3.35 (1.27)	3.35 (1.36)	3.00 (1.23)	2.50
Days per week of exercise adherence	1.37 (1.39)	1.31 (1.22)	1.42 (1.54)	1.52 (1.53)	0
Days per week of glucose monitoring	2.76 (2.17)	2.76 (2.20)	2.82 (2.14)	2.40 (1.92)	7
Days per week of foot care	1.79 (1.33)	1.72 (1.30)	1.76 (1.40)	2.11 (1.28)	0
MMQ contentment	34.5 (5.2)	34.5 (5.5)	34.7 (5.0)	33.2 (4.8)	42.0
MMQ ability	40.1 (7.4)	40.0 (7.3)	41.2 (7.6)	38.4 (7.6)	39.0
MMQ strategies	28.5 (7.5)	28.8 (7.2)	28.8 (7.5)	27.4 (8.5)	24.0
BDEFS time	10.1 (2.2)	10.0 (2.0)	10.4 (2.2)	9.2 (2.2)	8
BDEFS self-organization	9.6 (2.1)	9.8 (2.2)	9.4 (1.9)	9.2 (1.8)	10
BDEFS self-restraint	8.4 (2.2)	8.2 (2.3)	8.7 (2.2)	8.0 (2.0)	9
BDEFS motivation	8.5 (2.0)	8.6 (1.8)	8.4 (2.1)	8.3 (2.1)	9
BDEFS emotion	9.6 (2.1)	9.7 (1.8)	9.5 (2.6)	9.1 (1.7)	13
BDEFS total	46.2 (6.1)	46.6 (6.2)	46.6 (6.6)	44.0 (4.4)	49.0

Values are Mean (Standard Deviation) unless otherwise noted

Scores on the subscales of the MMQ and BDEFS-SF were calculated for all 120 participants (Table 2). MMQ ability and contentment scores fell in the middle range, indicating moderate subjective memory ability but moderate contentment with overall memory. Use of compensatory memory strategies (e.g., list making, reminders, etc.) was low. Scores on the BDEFS-SF were compared with a normative population and ranged from marginal clinical significance (76th–84th percentile) to markedly deficient (99th percentile) [8]. The mean score for the total BDEFS-SF placed these participants in the 94th percentile (mildly deficient). No significant differences were seen in scores between ethnic groups.

**Table 2:** Correlations between demographic characteristics and cognitive variables

	Age	Education	A1C	Years with DM
MMQ contentment	.09	-.07	-.19^b^	-.03
MMQ ability	.03	-.04	-.13	-.01
MMQ strategies	.07	-.09	-.01	-.05
BDEFS-SF time	.04	.09	.32^b^	.02
BDEFS-SF self-organization	.26^a^	.10	.12	.04
BDEFS-SF self-restraint	.08	.17	.34^a^	.19^b^
BDEFS-SF self-motivation	.03	.26^a^	.37^a^	-.03
BDEFS-SF self-regulation of emotion	-.01	-.05	.17	-.03
BDEFS-SF total	.14	.19^b^	.46^a^	.07

^a^Correlation is significant at the 0.01 level

^b^Correlation is significant at the 0.05 level

Pearson’s bivariate correlations (see Table 2) demonstrated a weak but significant positive correlation between age and self-organization on the BDEFS-SF (*r* = .26, *p* < 0.01) as well as between education and both BDEFS-SF self-motivation and total BDEFS-SF (*r* = .26, *p* < 0.01; *r* = .19, *p* < 0.05). Length of time with diabetes was positively correlated with decreased levels of self-restraint (*r* = .20, *p* < 0.05). Moderate significant correlations were found between A1C and some of the BDEFS-SF subscale scores (range, *r* = .32 to .46, *p* < 0.01). Total BDEFS-SF scores were weakly to moderately related to measures of diabetes self-management (range, *r* = -.07 to -.33, *p* < 0.01), and MMQ scores were weakly associated with diabetes self-management (Table 3).

**Table 3:** Correlations between diabetes self-management activities and cognitive variables

	Diet	Exercise	Glucose monitoring	Foot care
MMQ contentment	.09	-.03	-.01	-.02
MMQ ability	.25^a^	.23^b^	.00	.16
MMQ strategies	.12	-.02	-.10	-.01
BDEFS-SF time	-.17	.04	-.09	-.20^b^
BDEFS-SF self-organization	-.26^a^	-.14	-.22^b^	-.10
BDEFS-SF self-restraint	-.13	-.08	-.03	.71
BDEFS-SF self-motivation	-.22^b^	-.14	-.03	-.26^a^
BDEFS-SF self-regulation of emotion	-.17	.10	-.08	.29
BDEFS-SF total	-.33^a^	-.07	-.10	-.29^a^

^a^Correlation is significant at the 0.01 level

^b^Correlation is significant at the 0.05 level

Multiple linear regression analyses were conducted to predict the variation in quality of life and A1C. Variables significantly related to quality of life were entered simultaneously: MMQ ability, MMQ strategies, and BDEFS-SF total. Results showed that all variables in the model explained 38% of the perceived quality of life score and 23% of the A1C. Perceived memory ability (MMQ ability) was the strongest predictor of quality of life (β = .285, *p* = 0.004), and total scores on the BDEFS-SF were the strongest predictors of A1C (β = .445, *p* < 0.01).

To evaluate glycemic control, the same regression analysis was run, but A1C replaced the quality of life variables. The analysis showed that the cognitive variables, when considered together, also significantly explained 29% of the A1C. The strongest predictor in the model was the total BDEFS-SF score (β = .62, *p* = 0.011), followed by memory contentment (β = -.19, *p* < 0.05).

## Discussion

This study shows a clear relationship between perceived cognitive function, aspects of diabetes self-management, and glycemic control. The results also indicate that that the cognitive function variables examined here are important for health care providers to bear in mind when they discuss diabetes self-management with patients. This is consistent with prior studies that have demonstrated the importance of screening for executive dysfunction in people with diabetes, but how changes in executive function capacity impact self-management capacity needs more study [13–15].

Participants had some perceived impairment in memory and executive functioning, as indicated by the scores on the MMQ and BDEFS-SF. Both scales have been used to assess perceived memory and executive function in a number of groups [16, 17]. The MMQ has been tested, for example, in French (*n* = 114; mean age, 71.7) and Dutch (*n* = 294; mean age, 65.9) elderly non-clinical populations [6, 18]. Illman et al. tested it in a sample with temporal lobe epilepsy along with healthy controls [17]. All three studies found the MMQ valid and reliable. The present study is one of the first to use the MMQ and the BDEFS-SF for participants with T2DM. The results support the idea that people with T2DM have concern about their memory but use fewer strategies to compensate for their perceived memory difficulties. They also have lower levels of goal-directed behavior. In studies of other chronic conditions, people who judge their memory to be poor have also had difficulty in creating approaches to solving illness-related problems [19, 20]. Thus, people with diabetes and perceived memory difficulty may also have problems in developing strategies to deal with self-management issues. Complaints about memory should be considered by health care providers as an indicator of a potentially reduced ability to cope with diabetes problems. Of course there may be other contributors to the present results apart from diabetes alone, such as baseline intelligence, baseline brain volume, and genetic predispositions [21]. Future research is needed to investigate relationships between these cognitive function variables, objective neuropsychological tests, and functional MRI imaging.

No significant relationships were found between ethnicity and cognitive function variables in this study, but the higher prevalence of T2DM in minority populations reported in other studies indicates that the overall prevalence of cognitive dysfunction in those populations could be greater as well [22, 23]. In other words, there might still be issues in dealing with diabetes self-management that are not experienced by non-Hispanic whites. Previous research has shown a connection between cognition and diabetes in large populations; but there remain important differences in diabetes burden, and little is known about the relationships between diabetes and cognition in underserved groups [24].

Duration of diabetes has been associated with increased rates of cognitive impairment, with one study estimating that diabetes duration of greater than 10 years was linked to at least a 55% greater risk of major cognitive impairment than in people without diabetes [25]. The mean number of years with diabetes for those in the present study was 6.7 years, with a range of 1 to 24 years; 20% of the sample was in the >10 years category for cognitive impairment risk. Additionally, the number of co-morbidities is connected to an increased risk for cognitive impairment [25]. All participants except one in this sample had at least one co-morbidity. The most frequent co-morbidity was hyperlipidemia, followed by hypertension, both of which are conditions with well-established links to cognitive impairment [26].

Given our results and the association of higher A1C levels with lower perceived executive function, the possibility of an effect of cognition on diabetes self-management cannot be excluded. The presence of perceived impairment in executive function was significantly related to higher A1C levels. This is consistent with prior studies using objective, as opposed to self-report, measures of executive function [15]. The present study’s findings also support studies in which executive function, measured by objective neuropsychiatric tests, was related to lower levels of diabetes self-management [4, 15]. The present findings demonstrate that self-reports may be valid and useful tools to screen for cognitive dysfunction, comparable to tools that require specialized training to administer. Adherence to a dietary regimen for T2DM requires an individual to plan meals and be mindful of carbohydrate and calorie content while remembering other foods consumed that day. Such attention necessitates the utilization of both memory and executive function. Detailed data on the mechanisms of this effect of changes in memory and executive function on diabetes self-management are generally absent in current research [27].

Other investigations have shown that changes in cognitive function predicted diabetes medication adherence irrespective of treatment complexity [28]. The present results did not indicate a significant association between medication adherence and the examined cognitive variables. Some studies have examined mechanisms of the underlying pathology of cognitive impairment and diabetes such as fluctuations in glucose, but how those mechanisms might be clinically relevant remains unclear [5]. Although the present study was not undertaken to address the causal pathogenesis of cognitive decline, that topic should receive further investigation.

Both A1C and some of the examined cognitive variables were predictors of quality of life in this sample. Trials have shown that adults who engage in aerobic training show improvements in cognitive areas such as memory and executive function as well as in quality of life [29]. The present sample had low levels of exercise, and it is possible that interventions to increase physical activity could have improved their cognitive function, quality of life, and glycemic control. Other quality of life factors such as constricted life-space and few hours spent outside the home are also related to decreasing levels of cognitive function, but studies examining the effects of environmental factors affecting quality of life along with cognition and diabetes self-management have not been completed [30].

### Limitations

The results of this study show a relationship between perceived cognitive function, specifically executive function, and diabetes in a multiethnic sample. However, the study has some limitations. First, the study relied on self-reported data, which might include answers influenced by social desirability. In future studies, researchers might want to add neuropsychological tests in which participants are observed as they perform activities for comparison. Using lab-reported A1C from medical records or blood samples would also help to prevent this limitation. The small sample size from a single area of the country also limits generalizability. But the inclusion of a sample with over 50% from minority populations is a strength. Also, because of the study’s cross-sectional design, only associations were measured; conclusions about causality could not be made.

## Conclusions

Diabetes can affect cognitive function, and cognitive function may affect diabetes self-management, which is critical for glycemic control [5, 6]. Guidelines issued in the past several years have endorsed screening for cognitive impairment in people with diabetes because such impairment may hinder self-management [31, 32]. The results of this study suggest clinical implications: cognitive assessment of executive function and memory correlated with glycemic control should be included in diabetes treatment plans. Poor performance on self-report assessments for cognitive function can signal health care providers to ask patients about their perceived deficiencies in performing self-management tasks, so that they can develop individualized plans of care that take patients’ insights and preferences into account as well as refer patients for more in-depth neurological evaluations if needed [33]. Research regarding the processes that lead to cognitive changes in T2DM is needed; so too are studies of how those processes affect self-management activities. Finally, longitudinal studies should be conducted to examine the relationships between cognitive variables and self-management activities over time.

## Abbreviations

A1C: Hemoglobin A1C
BDEFS-SF: Barkley deficits in executive function scale –short form
MMQ: Multifactorial memory questionnaire
PI: Primary investigator
RAND SF-36: Rand short form health survey
SDSCA: Summary of diabetes self-care activities
T2DM: Type 2 diabetes
